# The Plant Virus Tomato Spotted Wilt Orthotospovirus Benefits Its Vector *Frankliniella occidentalis* by Decreasing Plant Toxic Alkaloids in Host Plant *Datura stramonium*

**DOI:** 10.3390/ijms241914493

**Published:** 2023-09-24

**Authors:** Zhijun Zhang, Jiahui Zhang, Xiaowei Li, Jinming Zhang, Yunsheng Wang, Yaobin Lu

**Affiliations:** 1 State Key Laboratory for Managing Biotic and Chemical Threats to the Quality and Safety of Agro-Products, Institute of Plant Protection and Microbiology, Zhejiang Academy of Agricultural Sciences, Hangzhou 310021, China; 15096100996zjh@gmail.com (J.Z.); lixiaowei1005@163.com (X.L.); zhanginsect@163.com (J.Z.); 2 Hunan Provincial Key Laboratory for Biology and Control of Plant Diseases and Insect Pests, Hunan Agricultural University, Changsha 410125, China; wyunsheng@gmail.com

**Keywords:** western flower thrips, *Frankliniella ocidentalis*, tomato spotted wilt orthotospovirus, *Datura stramonium*, alkaloids, tropane

## Abstract

The transmission of insect-borne viruses involves sophisticated interactions between viruses, host plants, and vectors. Chemical compounds play an important role in these interactions. Several studies reported that the plant virus tomato spotted wilt orthotospovirus (TSWV) increases host plant quality for its vector and benefits the vector thrips *Frankliniella occidentalis*. However, few studies have investigated the chemical ecology of thrips vectors, TSWV, and host plants. Here, we demonstrated that in TSWV-infected host plant *Datura stramonium*, (1) *F. occidentalis* were more attracted to feeding on TSWV-infected *D. stramonium*; (2) atropine and scopolamine, the main tropane alkaloids in *D. stramonium*, which are toxic to animals, were down-regulated by TSWV infection of the plant; and (3) *F. occidentalis* had better biological performance (prolonged adult longevity and increased fecundity, resulting in accelerated population growth) on TSWV-infected *D. stramonium* than on TSWV non-infected plants. These findings provide in-depth information about the physiological mechanisms responsible for the virus’s benefits to its vector by virus infection of plant regulating alkaloid accumulation in the plant.

## 1. Introduction

Many plant viruses exclusively rely on insect vectors for their transmission. These vector-borne plant viruses can change their host plant’s traits, thereby influencing the interactions between host plants and vectors [1,2,3,4,5,6,7]. However, the virus’s impact on the trophic system does not halt there. Previous studies have demonstrated that the virus-induced changes in host plant traits affect the competition among vector species [8,9] as well as the natural enemies of these vectors [10,11,12]. Infection by plant viruses typically leads to significant modifications in plant metabolism [13,14]. These alterations affect the primary metabolites [13,14,15,16] and the activation of defense responses, adjustments in the profiles of volatile secondary metabolites emitted by plants [3,17,18]. Consequently, these changes influence the performance of insect vectors and their ability to colonize host plants. The changes encompass not only volatile compounds used by foraging insects to locate suitable host plants but also the overall quality of the plant as a resource, involving variations in nutritional and defensive secondary compounds [3,5,6,7,15,17,18,19]. Such shifts in behavior and performance of insect vectors ultimately influence the transmission and epidemiology of the viruses they vectored [5,17,20,21,22,23]. While it is known that plant virus infections can lead to altering the profiles of non-volatile secondary metabolites in host plants [24,25,26], limited research has investigated the ecological significance of these non-volatile secondary metabolites within the interactions of this complex multitrophic system [27].

The western flower thrips (WFT), *F. occidentalis*, has emerged as a global invasive pest with far-reaching consequences, inflicting direct harm to plants through its feeding activities and indirect damage by serving as a vector for crop-infecting viral diseases like tomato spotted wilt orthotospovirus (TSWV) [28]. TSWV belongs to the *Orthotospovirus* genus, in the *Tospoviridae* family [29]. Thrips are exclusive vectors of orthotospoviruses. Although about 15 thrips species have been identified as vectors of orthotospoviruses, *F. occidentalis* is the most competent vector [29]. TSWV is transmitted by *F. occidentalis* in a persistent and propagative manner [30,31]. TSWV has become one of the most destructive plant viruses worldwide due to the vector, *F. occidentalis*, rapidly spreading across the world [32,33].

The weed *D. stramonium* plays an important role in the ecology and epidemiology of TSWV [34]. *F. occidentalis* adeptly acquires TSWV from *D. stramonium* and then transmits it to cultivated crops [34]. This facilitates a continuous cycle of disease transmission between *D. stramonium* and cultivated crops. Notably, it has been demonstrated that TSWV-infected plants remarkably expedite thrips development, making them more alluring to thrips for feeding and egg-laying compared to TSWV non-infected plants [35]. Specifically, *F. occidentalis* performs better on TSWV-infected plants than TSWV non-infected ones [1,22,36]. Within the host plants, the presence of TSWV triggers the activation of salicylic acid (SA) defense mechanisms, which in turn suppress the jasmonic acid (JA) defense response elicited by *F. occidentalis* [37]. The JA defense negatively influences *F. occidentalis* performance [38,39,40], as it governs the release of volatile compounds and acts as a deterrent to thrips feeding [18,40]. Nevertheless, the intricate molecular mechanisms and biochemical processes that underlie the positive impact of TSWV on *F. occidentalis* performance remain largely unexplored on the non-volatile metabolic level.

*F. occidentalis* derives advantages from the infection of host plants by TSWV, primarily because of the virus’s ability to trigger the SA response, which hampers the JA defense reaction. Nevertheless, within this intricate interplay, a diverse range of non-volatile secondary metabolites, such as alkaloids, hold significant nutritional value for the prosperity of various herbivorous insects, including thrips [41,42,43,44,45,46,47]. *D. stramonium* is rich in secondary metabolites, particularly tropane alkaloids like atropine (the racemic form of hyoscyamine) and scopolamine, which exhibit insecticidal properties [48,49]. We hypothesize that TSWV alters the non-volatile secondary metabolites, specifically alkaloids, to benefit its vector, *F. occidentalis*. This, in turn, results in an enhanced performance of *F. occidentalis* when it interacts with the TSWV-infected host plant. We thoroughly assessed the effects of TSWV infection on the host plant to fully understand how TSWV influences the performance of its vector by affecting the host plant, *D. stramonium*. These included the preference and population growth of *F. occidentalis*, along with identifying relevant changes in secondary metabolites (specifically alkaloids) induced by TSWV infection in *D. stramonium*. Furthermore, we conducted experiments on feeding behavior and survival with pure alkaloid compounds and identified alkaloid cues used by *F. occidentalis* to evaluate the plant quality.

## 2. Results

### 2.1. F. occidentalis Shows a Feeding Preference for TSWV-Infected D. stramonium

**Figure 1:**
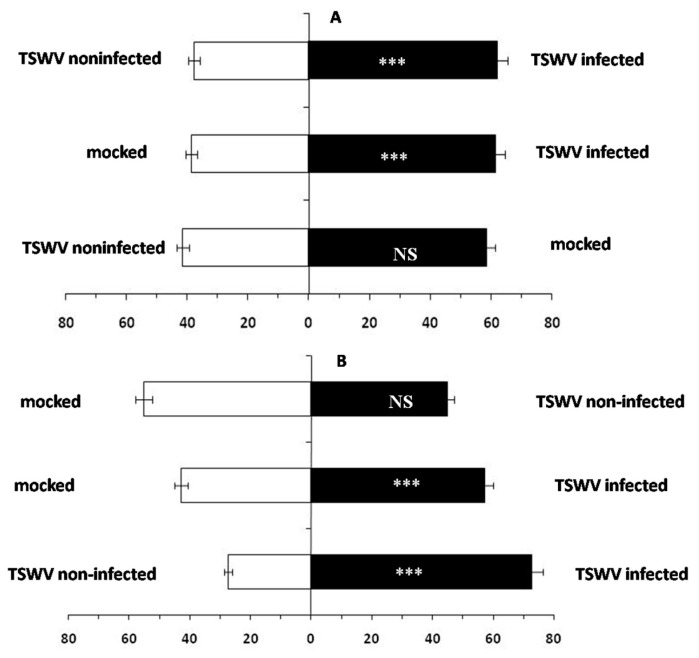
*F. occidentalis* feeding preference for TSWV-infected *D. stramonium*. (**A**) *F. occidentalis* larvae feeding choice using leaves and (**B**) *F. occidentalis* adult feeding choice using whole plants. Values are percentage of thrips that made a choice after 24 h where TSWV infected = *D. stramonium* treated and infected with TSWV, TSWV non-infected = *D. stramonium* with no treatment, and mock = *D. stramonium* treated with virus inoculation buffer but no virus. (Note: *** represents significant difference by the independent-sample *t* test at *p* < 0.01, NS = not significant.)

We investigated the indirect effect of TSWV infection on the feeding behavioral responses of the vector *F. occidentalis* using a two-choice assay involving TSWV-infected and TSWV non-infected leaves of *D. stramonium*, as well as entire TSWV-infected and TSWV non-infected *D. stramonium* plants. When thrips larvae were allowed to choose between TSWV-infected and TSWV non-infected leaves, approximately 60% of the larvae significantly preferred to feed on TSWV-infected leaves (*t* = 9.798, *df* = 4, *p* < 0.01, Figure 1A). In trials involving the TSWV-infected and TSWV non-infected whole plants and adult thrips, over 70% of the thrips were observed on the TSWV-infected plants (*t* = 7.317, *df* = 4, *p* < 0.01, Figure 1B). These results indicated that the vector *F. occidentalis* significantly prefers feeding on TSWV-infected *D. stramonium* over TSWV non-infected *D. stramonium* in both larval and adult stages.

### 2.2. TSWV Infection Reduces the Accumulation of Alkaloids in D. stramonium

**Figure 2:**
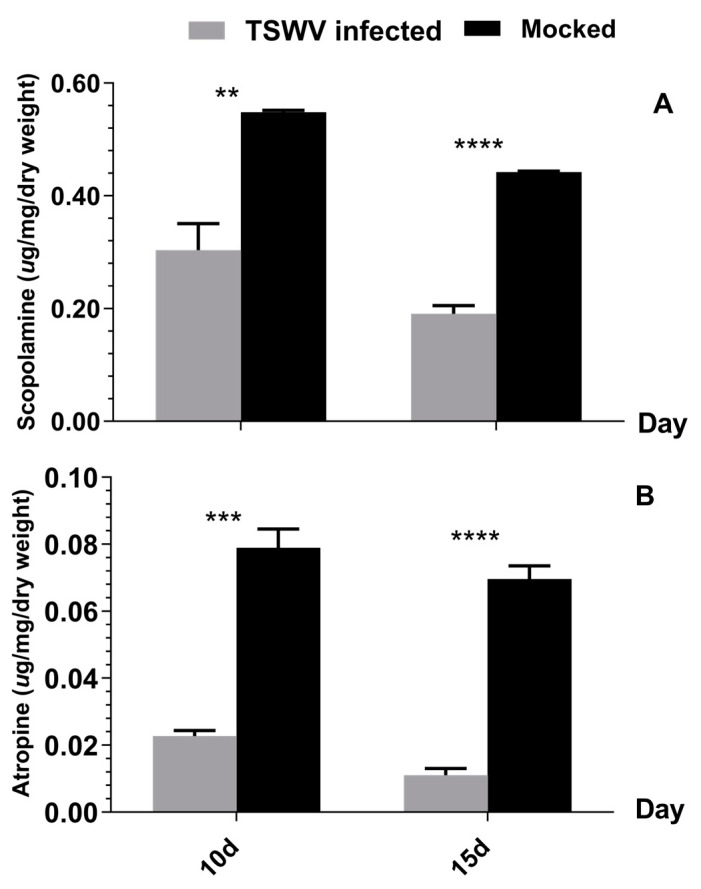
TSWV infection reduces the accumulation of tropane alkaloids in *D. stramonium*. (**A**) Level of scopolamine in mock (TSWV non-infected) *D. stramonium* (black) and TSWV-infected *D. stramonium* (gray); (**B**) level of atropine in mock (TSWV non-infected) *D. stramonium* (black) and TSWV-infected *D. stramonium* (gray). 10 d = 10 days after TSWV inoculation; 15 d = 15 days after TSWV inoculation. (Note: **, ***, **** represent significant difference by the independent-sample *t*-test at *p* < 0.01, *p* < 0.001, *p* < 0.0001, respectively.)

Non-volatile tropane alkaloids, such as scopolamine and atropine, present in *D. stramonium*, act as toxins against herbivores, often triggering a repellent effect [48,50,51,52]. We investigated the alterations of tropane alkaloids (scopolamine and atropine) levels in TSWV-infected *D. stramonium*. The concentrations of scopolamine were 0.304 μg/mg and 0.191 μg/mg in TSWV-inoculated *D. stramonium* after 10 d and 15 d, respectively. These values were notably lower than those in TSWV non-infected samples, which exhibited scopolamine concentrations of 0.548 μg/mg and 0.442 μg/mg after 10 d (*t* = 5.181, *df* = 4, *p* < 0.01) and 15 d (*t* = 17.664, *df* = 4, *p* < 0.0001), respectively (Figure 2A). Atropine concentrations followed a similar trend, at 0.023 μg/mg and 0.011 μg/mg in TSWV-inoculated *D. stramonium* after 10 d and 15 d, respectively. In contrast, significantly higher concentrations of 0.079 μg/mg (*t* = 9.790, *df* = 4, *p* < 0.001) and 0.070 μg/mg (*t* = 13.420, *df* = 4, *p* < 0.0001) were observed in TSWV non-infected *D. stramonium* at 10 d and 15 d, respectively (Figure 2B).

### 2.3. Alkaloid Interactions with F. occidentalis: Repellence and Toxicity

#### 2.3.1. Repellent Effect of Scopolamine and Atropine on *F. occidentalis*

**Figure 3:**
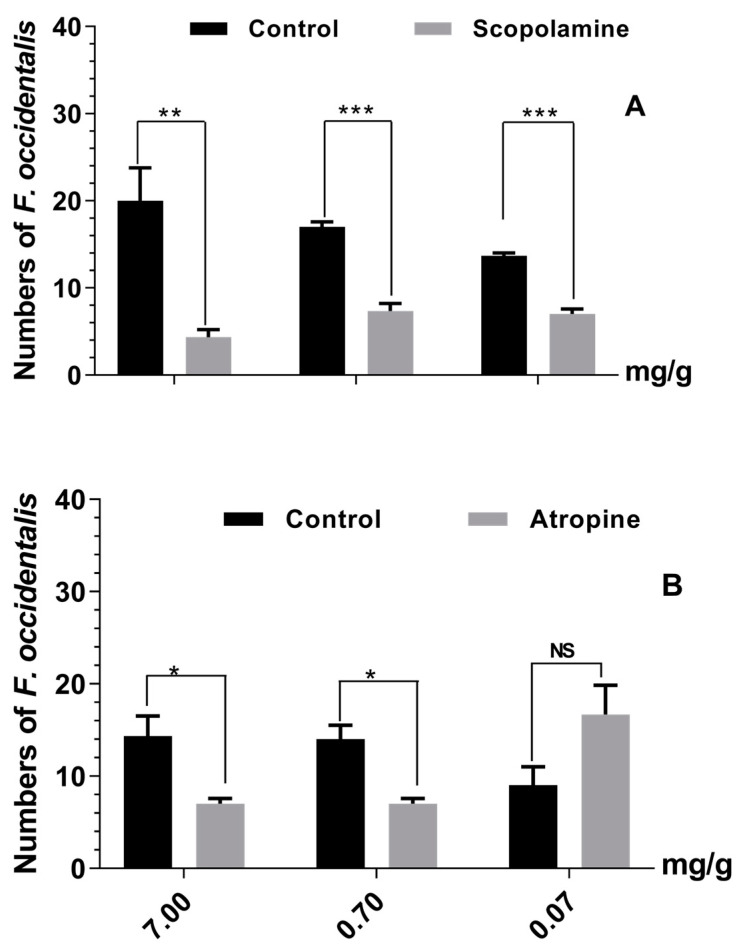
Effect of tropane alkaloids on *F. occidentalis* feeding choice in a Y-tube. *F. occidentalis* were presented with feeding solution (30 mg/g sucrose) on one arm containing tropane alkaloids (**A**) scopolamine or (**B**) atropine at 7, 0.7, or 0.07 mg/g, respectively. The other arm contained the 30 mg/g sucrose solution without tropanes (control) accordingly. Values are mean number of thrips following three replicates ±SEM, *n* = 3. (Note: *, **, *** represent significant difference by the independent-sample *t* test at *p* < 0.05, *p* < 0.01, *p* < 0.001, NS = not significant, respectively.)

In our investigation of tropane alkaloid effects on *F. occidentalis* feeding choice, we conducted choice experiments utilizing scopolamine and atropine-principal tropane alkaloids found in both TSWV-infected and TSWV non-infected *D. stramonium*. Adult *F. occidentalis* were offered the option of feeding on a 30 mg/g sucrose solution containing tropane (treatment) and devoid of it (only 30 mg/g sucrose) through a Y-tube setup. Notably, our findings revealed that the solutions containing tropane alkaloids (scopolamine and atropine) had a repellent effect on thrips feeding, except observed at 0.07 mg/g atropine concentration (Figure 3). These findings supported our hypothesis that tropane alkaloid, scopolamine, serve as deterrents for *F. occidentalis*, potentially contributing to the *F. occidentalis* preference for TSWV-infected *D. stramonium* over their TSWV non-infected counterparts.

#### 2.3.2. Toxicity of Scopolamine and Atropine to *F. occidentalis*

The validity of scopolamine and atropine toxic to the *F. occidentalis* was established through an in vitro bioassay conducted at 7 mg/g, 0.7 mg/g, and 0.07 mg/g concentration. Our findings indicated a significant mortality elevation across all concentrations when compared to the negative control 30 mg/g sucrose concentration (Table 1). The mortality of thrips attributed to scopolamine and atropine exhibited a positive correlation with the concentration of these two metabolites. Furthermore, a collective analysis revealed a noteworthy reduction in thrips mortality as the concentration of the two metabolites decreased.

### 2.4. Performance of F. occidentalis on TSWV-Infected or TSWV Non-Infected D. stramonium

The longevity of both male and female *F. occidentalis* on TSWV-infected *D. stramonium* was significantly extended compared to those on TSWV non-infected *D. stramonium* plants. Collectively, the average adult longevity on TSWV-infected *D. stramonium* (7.30 d) exhibited a significant increase over that on TSWV non-infected *D. stramonium* (5.98 d) (Table S1). Moreover, the mean total oviposition period of *F. occidentalis* on TSWV-infected *D. stramonium* plants (4.39 d) surpassed that on TSWV non-infected *D. stramonium* plants (3.31 d). Additionally, the mean fecundity of females, recorded at 17.47 larvae per female, was significantly elevated on TSWV-infected *D. stramonium*, in contrast to the mean fecundity of 5.40 larvae per female on TSWV non-infected *D. stramonium* (Table S2).

The female age-specific fecundity (*f_xj_*, where x represents age, and j represents stage) and the total-population age-specific maternity (*m_x_*) of *F. occidentalis* on TSWV-infected *D. stramonium* plants were significantly higher than those on TSWV non-infected *D. stramonium* (Figure S1). TSWV infection of *D. stramonium* significantly impacted on the life table parameters of the *F. occidentalis* feeding on TSWV-infected *D. stramonium*. Specifically, TSWV infection influenced the intrinsic rate of increase (*r_m_*), the finite rate of increase (*λ*), and the net reproductive rate *R*_0_ of *F. occidentalis* feeding on TSWV-infected *D. stramonium*, which were significantly higher than those of *F. occidentalis* feeding on TSWV non-infected *D. stramonium*. When feeding on TSWV-infected *D. stramonium*, the *r_m_*, *λ*, and *R*_0_ values for *F. occidentalis* were 0.178, 1.58, and 40.32, respectively. However, the *r_m_*, *λ*, and *R*_0_ values for *F. occidentalis* were 0.013, 1.09, and 11.11, respectively (Table 2), when feeding on TSWV non-infected *D. stramonium*.

**Figure 4:**
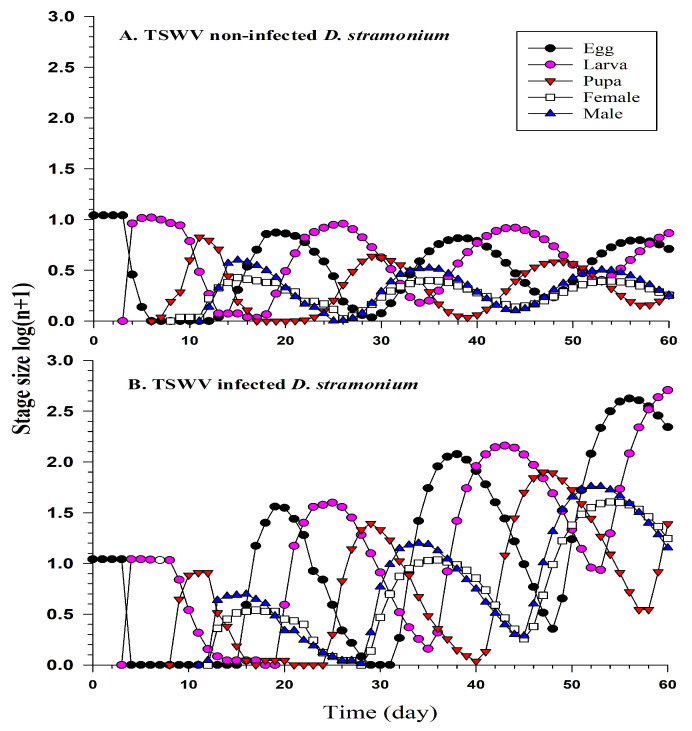
Population projection of *F. occidentalis* on TSWV non-infected *D. stramonium* (**A**) and TSWV-infected *D. stramonium* (**B**).

Population projections indicated that *F. occidentalis* would experience more rapid growth on TSWV-infected *D. stramonium* compared to TSWV non-infected plants. Starting with 100 eggs, the projected population size on TSWV-infected *D. stramonium* would exceed 5000 individuals after 60 days, in contrast, the population size of *F. occidentalis* on TSWV non-infected *D. stramonium* would only reach around 300 individuals within the same time frame (Figure 4).

## 3. Discussion

Our study highlights the crucial role of alkaloids in mediating interactions between vectors and viruses. To our knowledge, this is the first instance where virus infection has been demonstrated to enhance the performance and expedite the population growth of its insect vector by reducing alkaloid accumulation in the host plant. Previous studies have indicated that TSWV benefits *F. occidentalis* by activating the host plant’s SA signal pathway and inhibiting the JA signal pathway, resulting in decreased volatile terpene [18], which has a negative effect on *F. occidentalis* [37,38,39,53]. The induction of the JA signal pathway contributes to the production of alkaloids and other secondary metabolites in plants [18,50]. TSWV infection in *D. stramonium* may hinder the biosynthesis of these alkaloids (scopolamine and atropine) by promoting the SA pathway, which opposes the JA signal pathway. Further exploration is required to unveil the precise underlying mechanisms. An analogous interaction is observed in Ageratum enation virus (AEV), where AEV down-regulates the biosynthesis of morphine alkaloids in its host, the opium poppy (*Papaver somniferum* L.) [26]. This phenomenon also influences the defense response of host plants to plant viruses [14], potentially enhancing transmission at both the virus and vector levels. These findings imply that TSWV infection of the host plant impacts the vector *F. occidentalis* beyond modulating plant signaling pathways. It also has the potential to alter plant secondary metabolism.

Previous studies have revealed the function of specific alkaloids against thrips [42,44,45,46,54,55]. These plant-derived alkaloids serve as potent toxins or repellents against thrips, demonstrating constitutive and inductive properties within host plants that help plants withstand pest attacks [45,46,56]. Notably, in the case of tomatoes, a solanaceous species similar to *D. stramonium*, non-volatile alkaloids have demonstrated their efficacy in repelling thrips [51,57]. The non-volatile tropane alkaloids prevalent in *D. stramonium*, including scopolamine and atropine, have also demonstrated toxicity against various herbivores [48,49,50,52]. Consequently, it is reasonable to infer that scopolamine and atropine, synthesized by *D. stramonium*, are likely to exhibit repellent or toxic properties when faced with thrips. Furthermore, our study highlighted that scopolamine and atropine alkaloids exhibited heightened concentrations in TSWV non-infected *D. stramonium* but experienced a significant decrease in TSWV-infected counterparts (Figure 2). These compounds at the most experimental concentration exhibited a deterrent effect or exerted direct toxicity to thrips during feeding (Figure 3, Table 1). While the atropine at low concentration (0.07 mg/g) did not significantly enhance thrips’ feeding preference, the effects of the atropine on thrips’ feeding preference at lower concentrations need to be further determined. Notably, TSWV infection decreased tropane alkaloid levels, which likely explains the observed improvements in *F. occidentalis* performance during their development on TSWV-infected plants (Table 2).

The AEV infection of the host plant down-regulates the morphine alkaloid biosynthesis pathway [26], grapevine red-blotch-associated virus (GRBaV) infection inhibits the phenylpropanoids pathway [24]. However, the exact mechanisms by which plant virus infection reduces the synthesis of alkaloids and other secondary metabolites remains unknown. Tropane alkaloid, scopolamine and hyoscyamine, biosynthetic pathways are involved in polyamine metabolism in the early steps. Putrescine is a common precursor of both polyamines, such as spermidine and spermine, and tropane alkaloids [58]. Tropane alkaloid biosynthesis begins with the methylation of putrescine to N-methyl-putrescine by *putrescine N-methyltransferase* (*PMT*), followed by the oxidative deamination of N-methyl-putrescine to 4-methylaminobutanal with *N-methyl-putrescine oxidase* (*MPO*). The central intermediate N-methyl-1-pyrrolium cation for tropane alkaloid biosynthesis results from the spontaneous cyclization of 4-methylaminobutanal. The condensation of N-methyl-1-pyrrolium cations with acetoacetic acid results in hygrine converted to tropinone. The reduction of tropinone catalyzed by *tropinone reductase 1* (*TR 1*) forms tropine, and the tropine condenses with the phenylalanine-derived (*R*)-phenyl-lactate by *littorine synthase* (*LS*) to yield littorine. Littorine undergoes rearrangement to hyoscyamine aldehyde by a *cytochrome P450* (*Cyp80F1*). The hyoscyamine aldehyde is converted to hyoscyamine following an unknown process. After that, hyoscyamine is epoxidated and yields the end product, scopolamine, via a two-step reaction: 6β-hydroxylation of the tropane ring followed by intra-molecular epoxide formation that is catalyzed by a 2-oxoglutarate-dependent dioxygenase, *hyoscyamine 6β-hydroxylase* (*H6H*) [59,60,61]. The *PMT* and *H6H* are the rate-limiting upstream and downstream enzymes in the biosynthesis of tropane alkaloids (hyoscyamine and scopolamine), which catalyze the first and last steps of the process, respectively [58]. How does TSWV infection affect the production of tropane alkaloids? Further research is required to determine the relationship between the signal or transcriptional regulation of the tropane alkaloid biosynthesis pathway and the TSWV-induced inhibition of tropane alkaloid biosynthesis.

## 4. Materials and Methods

### 4.1. Plants, Virus Isolates, and Insect Populations

**Figure 5:**
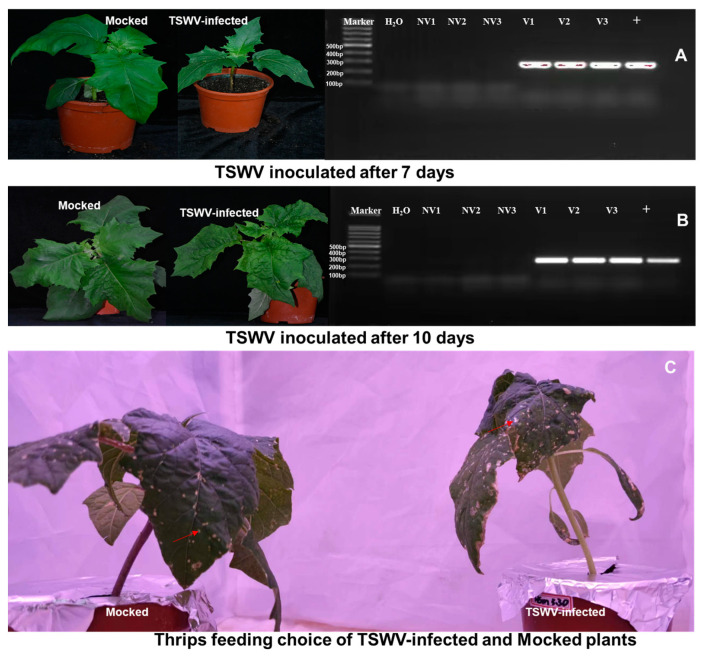
The symptom of tomato spotted wilt orthotospovirus (TSWV)-infected *D. stramonium* ((**A**) right; (**B**) right) and *F. occidentalis* feeding on mock and TSWV infected *D. stramonium* (**C**). Note: Infected leaves were ground in 0.05 M phosphate buffer (pH 7.0) and applied to the host plant using a soft finger-rubbing technique. Infected plants were tested at 7 ((**A**) left) and 10 ((**B**) left) dpi by RT-PCR. Object band size 273 bp from N protein of TSWV, primers. PTSW-F: GGGTCAGGCTTGTTGAGGAAAC; PTSW-R: TTCCCTAAGGCTTCCCTGGTG. NV = mocked; V = TSWV-infected; H_2_O = negative; + = positive. The number of feeding scars (red arrow) on the plant represents *F. occidentalis* feeding preference (**C**).

Plants, virus isolates, and insect populations were maintained according to a previous study [62]. *D. stramonium* plants were grown and maintained in thrips-proof screen-cages under greenhouse conditions. To achieve systemic virus infection, plants at the three-true-leaf stage were mechanically inoculated with TSWV-YN isolates that were originally collected in Yuxi County, Yunnan Province, China [63], and maintained by thrips-mediated passage on potted *D. stramonium*. Only plants with obvious symptoms (chlorotic, curling, and spotted leaves) were selected, and infection was confirmed by molecular detection using reverse transcription–polymerase chain reaction (RT-PCR) approximately 7 days and 10 days after inoculation (Figure 5). The control *D. stramonium* plants were mock-inoculated with a buffer having no virus at the same time. The *F. occidentalis* colony was collected in Beijing, China, in 2003 and grown on fresh green bean (*Phaseolus vulgaris*) pods in a climate-controlled chamber (27 ± 1 °C, 16L:8D) [64].

### 4.2. Thrips’ Preference for TSWV-Infected and TSWV Non-Infected Plants

#### 4.2.1. Preference of Larvae Thrips

According to previously described methods [55] with some modifications, the second-stage larvae of *F. occidentalis* were offered a choice between two detached leaves: one from TSWV-infected *D. stramonium* and the other from TSWV non-infected *D. stramonium*. The two leaves were placed on opposite sides of a Petri dish (15 cm diameter) and covered with moist filter paper approximately 5 cm apart from each other. The position of the leaves was alternated between replicates. Forty second-stage larvae of *F. occidentalis* were transferred into each dish in the gap between the two leaves and were allowed to feed overnight. After 24 h, the number of larvae on each leaf was counted.

#### 4.2.2. Preferences of Adult Thrips

Adult *F. occidentalis* were offered a choice between a TSWV-infected and an TSWV non-infected *D. stramonium* plant in thrips-proof screen-cages (50 cm × 50 cm × 50 cm). The two plants were placed opposite each other in the cage, approximately 20 cm apart. The position of the plants was rotated between replicates. Fifty adult *F. occidentalis* were transferred to each cage in the gap between the two plants and allowed to feed overnight. After 24 h, the number of adults on each plant was counted.

### 4.3. Analysis of Alkaloids Scopolamine and Atropine

Leaf tissue for alkaloid analysis was collected from TSWV-infected *D. stramonium* and TSWV non-infected *D. stramonium*. Plant samples were ground under liquid N_2_ using a cold mortar and pestle, and dried by lyophilization, then stored in paper bags until analysis. The alkaloids scopolamine and atropine were extracted and analyzed according to previously described methods [65]. Plant powder (100 mg) was weighed into volumetric flasks. Extraction was ensured after 30 min. Ultrasonication was conducted after adding 50 mL of solvent mixture (CH_3_OH: H_2_O = 3:2 (*v*/*v*)) to each sample. The extracts were purified by centrifugation (15 min at 10,000 min^−1^) and filtration through a 0.22 μm pore size syringel-free filter (Agela). Samples were stored in the dark at 4 °C until the LC-MS analysis was carried out.

The LC–MS system consisted of a liquid chromatograph (Surveyor Autosampler Plus and Accela Pump) and an MS detector with electrospray ion source and quadrupole analyzer (TSQ Quantum Ultra, Thermo, Waltham, MA, USA). LabSolutions software TargetQuan 3 (Thermo) was used to control the LC–MS system and for data processing. Chromatographic separations were performed on a 00D-4462-E0 Kinetex C18 column (100 mm × 4.6 mm, 2.6 μm, Phenomenex, Torrance, CA, USA). A gradient of mobile phase A (methanol) and mobile phase B (5 mmol/L ammonium acetate and 0.1% (*v*/*v*) formic acid in methanol) was used for the separations. The gradient profile was set as follows: 0.00 min 5% A eluent, 1.00 min 5% A eluent, 3.00 min 95% A eluent, 5.00 min 95% A eluent, 5.01 min 5% A eluent, and 7.00 min 5% A eluent. The flow rate was 0.6 mL min^−1^, and the column temperature was 35 °C. The injection volume was 20 μL for *D. stramonium* extracts and standard mixtures. The ions of the compounds and their retention times were as follows: *m*/*z* 304.2 and 2.42 min for scopolamine and *m*/*z* 290.0 and 2.62 min for atropine. The electrospray source was operated in positive mode, and the interface conditions were as follows: a capillary voltage of 3.5 kV, a capillary temperature of 350 °C, and a vaporizer temperature of 200 °C.

The quantification of atropine and scopolamine was performed using standard solutions. From the methanolic stock solutions of atropine and scopolamine (1 mg mL^−1^ each), calibration solutions were prepared in the concentration range of 10^−6^ to 10^−2^ mg mL^−1^ by dilution of the stock solution with solvent mixture (1% (*v*/*v*) formic acid in methanol: water = 7:93). The solutions were stored at 4 °C and used for one week.

### 4.4. Effects of the Alkaloids Scopolamine and Atropine on Thrips’ Preference and Survival

#### 4.4.1. *F. occidentalis* Choice between Feeding Solutions

According to previously described methods [55], 40 adults of *F. occidentalis* were placed in a glass Y-tube closed at the lower end. Both upper arms were covered with a Parafilm membrane, and 125 μL of 30 mg/g sucrose solution was applied to one side and covered with a second Parafilm layer as a control. The 125 μL of sucrose solution (30 mg/g) used to cover the other side contained 7 mg/g, 0.7 mg/g, or 0.07 mg/g scopolamine or atropine that was added to the feeding solution. *F. occidentalis* can pierce through the Parafilm membrane and ingest the feeding solution by sucking, similar to their feeding habits on natural sources. After 24 h, *F. occidentalis* residing on both sides of the Parafilm membrane were counted. The experiment was repeated three times for each concentration of scopolamine and atropine.

#### 4.4.2. *F. occidentalis* Survival on Feeding Solutions

Second-instar *F. occidentalis* were obtained from the lab population. The bioassay was performed according to the methods of a previous study [44]. Wells of 96-well plates were filled with 50 μL of test solution. The dilution was a 30 mg/g sucrose solution (in distilled water). The blank control was a 30 mg/g sugar solution in water. There was one negative/positive controls for each group: wells containing water to verify that western flower thrips larvae could not survive without eating food and a solution containing the insecticide abamectin (1 mg/kg) to have an absolute positive control to compare to the effect of alkaloids. All treatments (i.e., one blank control, six treatments with three different alkaloid concentrations of a single scopolamine and atropine, and one negative/positive controls) were placed in 96-well plates. Each treatment consisted of four columns of 96-well plates, and each column contained 8 wells as a replicate. Each treatment contained 32 thrips. The second-instar *F. occidentalis* was individually transferred to each cup of an 8-cup strip. The cup was sealed with Parafilm and placed on top of the 96-well plates and then turned upside down. *F. occidentalis* were then allowed to feed on the test solutions provided. The plates were randomly placed in a growth chamber with standard *F. occidentalis* rearing conditions (L:D, 16:8, 27 ± 1 °C). After five days, the number of surviving larvae was counted under a stereomicroscope. Mortality was calculated as the number of dead larvae in each replicate of the four replicates. In single-alkaloid experiments, scopolamine and atropine were tested at the following concentrations: 7 mg/g, 0.7 mg/g, and 0.07 mg/g.

### 4.5. Thrips Performance Experiments

#### 4.5.1. Development and Juvenile Survival

Eggs were collected by placing fresh leaves of TSWV-infected and TSWV non-infected *D. stramonium* (with moist cotton wool wrapped around the leaf stalk) in *F. occidentalis* rearing glass jars containing hundreds of adults. Female adults were allowed to oviposit on the leaves for 12 h. The adults were removed, and the leaves with eggs were transferred into two different Petri dishes (15 cm diameter), which were then placed in a cabinet set at 27 ± 1 °C and with a 16:8 (L:D) photoperiod until the eggs hatched. For developmental investigations, leaves were collected from TSWV-infected and TSWV non-infected plants, and leaf discs (1.5 cm diameter) were removed from the leaves using a cork borer. Each disc was placed in a plastic Petri dish (4 cm diameter) filled with moist filter paper. About 100 newly hatched larvae in each treatment were individually transferred to the Petri dishes (4 cm diameter, each Petri dish with one individual constituted a replicate) using a fine hairbrush when the egg hatched. Each Petri dish was sealed with a Parafilm membrane to prevent the escape of *F. occidentalis*. Leaf discs were changed daily. The development and survival of each juvenile were assessed daily until the larvae died or matured.

Four developmental stages of the insect were assessed. Since the eggs were laid inside the leaf and were not visible, the duration of the egg stage was recorded as the time when the leaves with newly laid eggs were placed in the jars until the time when larvae emerged on the leaf surface [66]. The first- and second-instar larval stages were combined, because there are no obvious morphological differences between the two stages except for size. The prepupa is recognized by the short wing sheaths and erect antennae; the pupa has long wing sheaths that almost reach the end of the abdomen, and the antennae are bent backwards along the head. Although there are obvious morphological differences between the prepupal and pupal stages, the prepupal and the pupal stages were also combined into one stage, because both stages share the same characteristics of not eating or moving unless they are disturbed. In addition, the duration of the prepupal stage was not more than one day. Adults were identified by the emergence of wings.

#### 4.5.2. Adult Survival and Oviposition

At adult emergence, about 30 pairs of newly emerged female and male adults from each treatment were placed in a glass cylinder (30 mm diameter × 40 mm height, one pair per glass cylinder constituting one replicate) containing a fresh *D. stramonium* leaf disc as described in “Development and juvenile survival”. The two ends of the glass cylinders were sealed with a Parafilm membrane. The leaf discs were replaced daily, and the replaced discs were individually transferred to Petri dishes (4 cm diameter), and the lids were sealed with Parafilm. The live adults of each sex were recorded until the adults died. The number of the first instars was counted and used to represent the fecundity of females [64], as thrips eggs were laid below the leaf surface and could not be easily observed [67]. The number of first instars per female per day, first instars per female per lifetime, and adult longevity were recorded.

### 4.6. Data Analysis

The analysis of raw thrips performance data adhered to established methodologies [68]. The key population growth parameters, encompassing the intrinsic rate of age-specific survival rate (*l_x_*), age-specific maternity (*m_x_*), the age-specific fecundity (*f_xj_*), intrinsic rate of increase (*r_m_*), net reproductive rate (*R*_0_), finite rate of increase (*λ*), mean generation time (*T*), life expectancy (*e_xj_*), and reproductive value (*v_xj_*) were examined using age-stage, two-sex life table method by TWOSEX-MSChart program [69,70,71]. For enhanced precision in estimations, means and standard errors of life table parameters were obtained through a bootstrap procedure involving 100,000 replicates (*m* = 100,000). The paired bootstrap test [72] was used to compare the differences of developmental time, adult longevity, adult pre-oviposition period, total pre-oviposition period, oviposition days, and fecundity of *F. occidentalis* between TSWV-infected and TSWV non-infected *D. stramonium*. Comparisons of population parameters (*r_m_ λ*, *R*_0_, and *T*) between the two treatments were executed using the paired bootstrap test [73,74], leveraging the confidence interval of differences [72]. Survival rates and fecundity data were utilized for projecting population growth using TIMING-MSChart program [75], following established methodologies [76]. Thrips’ preference for TSWV-infected and TSWV non-infected *D. stramonium*, the effects of alkaloids (scopolamine and atropine) in *D. stramonium* on thrips’ preference, and the distinction in alkaloid (scopolamine and atropine) concentrations between TSWV-infected and TSWV non-infected *D. stramonium* were analyzed through a *t*-test conducted using the SPSS software package (ver.17, SPSS Inc., Chicago, IL, USA). The mortality of thrips exposed to different tropane alkaloid treatments was analyzed by a one-way analysis of variance (ANOVA) Tukey’s multiple test. The resulting data were visualized using GraphPad Prism version 7.00 and Sigma plot 10.0.

## 5. Conclusions

The present study demonstrated that TSWV infection of *D. stramonium* causes significant reduction of repellent and toxic tropane alkaloids in *D. stramonium*, the common host plant of TSWV, and its thrips vector *F. occidentalis*. The thrips vector *F. occidentalis* can prefer and perform better on TSWV-infected plants than on TSWV non-infected ones, which should increase the number of vectors and the success of virus transmission. To our knowledge, this is the first time that the virus benefitting itself and its vector by modulating alkaloids metabolism in the common host plant has been demonstrated. This study provides an insight into the mechanism by which TSWV benefits its vector, western flower thrips. Moreover, these findings provide in-depth information to understand the interactions among plant viruses, vectors, and their host plants.

## Figures and Tables

**Table 1:** The mortality of *F. occidentalis* exposed to different concentrations of tropane alkaloids.

Treatment (mg/g)	Atropine (%)	Scopolamine (%)	Atropine + Scopolamine (%)
CK	16.04 ± 1.09 a		
0.07	35.18 ± 2.73 b	36.79 ± 2.43 b	42.05 ± 2.54 b
0.7	40.63 ± 4.65 b	44.79 ± 3.91 b	43.75 ± 2.88 b
7	64.06 ± 5.99 c	66.07 ± 3.54 c	62.50 ± 5.55 c
Negative	77.08 ± 4.31 c		
Positive	94.79 ± 2.41 d		

Note: Values are mean percentage of thrips dead caused by exposure to different treatments. Mean values within the same column followed by different letters are significantly different by a one-way analysis of variance (ANOVA) Tukey’s multiple test (*p* ≤ 0.05). CK represents 30 mg/g sucrose solution without tropanes; 0.07, 0.7, and 7 represents 30 mg/g sucrose solution contained tropanes 0.07 mg/g, 0.7 mg/g, and 7 mg/g, respectively; Negative represents only water; Positive represents only water containing the insecticide abamectin (0.001 mg/g).

**Table 2:** Life table parameters of *F. occidentalis* on TSWV-infected and non-infected *D. stramonium* leaves.

Treatment	*N*	Intrinsic Rate of Increase *r_m_* (/d^−1^)	Finite Rate of Increase *λ* (/d^−1^)	Net Reproduction Rate *R*_0_ (Offspring/Individual)	Mean Generation Time *T* (d)
TSWV non-infected	107	0.013 ± 0.017 b	1.09 ± 0.018 b	11.11 ± 0.33 b	18.24 ± 2.06 a
TSWV-infected	93	0.178 ± 0.013 a	1.58 ± 0.014 a	40.32 ± 0.96 a	18.39 ± 0.17 a

Note: mean values within the same column followed by different letters are significantly different determined by the paired bootstrap test with 100,000 resampling (*p* ≤ 0.05).

## Data Availability

No new data were created or analyzed in this study. Data sharing is not applicable to this article.

## Supplementary Materials

The following supporting information can be downloaded at: https://www.mdpi.com/article/10.3390/ijms241914493/s1.
