# Supplementary material for: The Plant Virus Tomato Spotted Wilt Orthotospovirus Benefits Its Vector Frankliniella occidentalis by Decreasing Plant Toxic Alkaloids in Host Plant Datura stramonium
Source: Int J Mol Sci. 2023 Sep 24;24(19):14493. doi: 10.3390/ijms241914493 (PMC10572871; doi:10.3390/ijms241914493)
Supplement: Supplementary file 1 [file ijms-24-14493-s001.zip › Supplementary Figure S1.pdf]

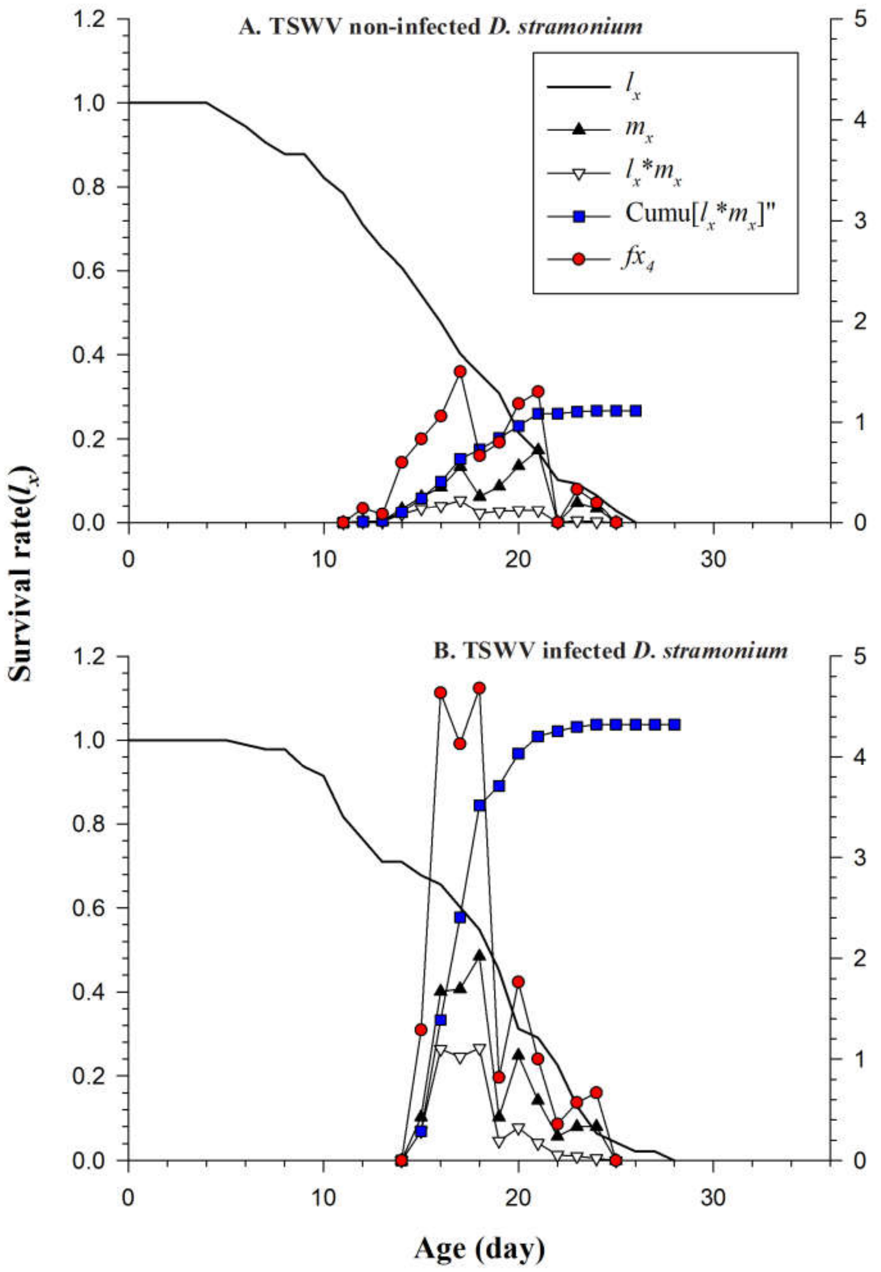

Figure S1: Age-specific survival rate and fecundity versus age of *F. occidentalis* on TSWV non-infected *D. stramonium* (A) and TSWV-infected *D. stramonium* (B)
